# “Evolution” of intravascular leiomyomatosis

**DOI:** 10.1186/s12905-023-02618-3

**Published:** 2023-09-11

**Authors:** Li Chen, Yunping Yang, Chengzhi Zhao

**Affiliations:** 1 Department of Obstetrics and Gynecology, Chongqing Health Center for Women and Children, Women and Children’s Hospital of Chongqing Medical University, No.120 Longshan Road, Yubei District, Chongqing, People’s Republic of China; 2 Department of Quality Management, Chongqing Health Center for Women and Children, Women and Children’s Hospital of Chongqing Medical University, No.120 Longshan Road, Yubei District, Chongqing, People’s Republic of China

**Keywords:** Intravascular leiomyomatosis, Hysteroscopic myomectomy, Laparoscopic surgery

## Abstract

**Background:**

Intravenous leiomyomatosis (IVL) is a rare and specific type of smooth muscle tumor that is histologically benign but has a malignant biological behavior. It is commonly associated with a history of uterine leiomyomas.

**Case presentation:**

A 36-year-old woman, G1P1, presented to the hospital with left lower abdominal pain for 2 months and she has accepted hysteroscopic myomectomy about 1 year ago. Ultrasound venography, echocardiography and computed tomography venography (CTV) of inferior vena cava were performed, which revealed IVL located in left intramural myometrium walls growing along the left ovarian vein reaching the level of the lumbar 5-sacral 1 disc. Laparoscopic bilateral salpingo-oophorectomy and hysterectomyis were scheduled. The IVL in the left ovarian vein and parauterine venous plexus were detected and excised completely during surgery. IVL was diagnosed by postoperative pathology and immunohistochemistry. The patient recovered well after surgery. No surgical-related or anesthesia-related complications occurred.The 3-month follow-up CTV of inferior vena cava and echocardiography examination revealed normal.

**Conclusions:**

The cause of IVL is unknown, this observation demonstrates that hysteroscopic myomectomy might lead to the occurrence of IVL.

## Background

Intravenous leiomyomatosis (IVL) is a rare and specific type of smooth muscle tumor that is characterized by the presence of vascular extension and invasion of benign smooth muscle lesions in a worm-like manner into the pelvic and systemic vasculature system [1, 2]. The incidence of IVL is low and the specific pathogenesis is still unclear. However, most studies believe that the tumor originates in the uterus, spread along the intra- and extrauterine venous lumen and can extend to the inferior vena cava, right atrium, right ventricle, and pulmonary artery [3]. About 83.5–94.3% of IVL patients had uterine fibroids or previous history of uterine fibroid surgery as reported [4, 5]. This disease may remain hidden and the clinical manifestations in early-stage patients are non-specific and it is easy to be misdiagnosed or missed. Since IVL is aggressive and it’s atypical symptoms, early recognition and management can prevent fatal consequences. Patients with a history of uterine fibroids or surgery should request scheduled follow-up for early detection and treatment.

## Case presentation

A 36-year-old woman, G1P1, presented to the hospital with left lower abdominal pain for 2 months. She had accepted hysteroscopic myomectomy for menorrhagia and anemia caused by submucosal fibroid (FIGO classification:type 1, measuring 4 cm in diameter by ultrasonography) about 1 year ago. During the operation, the fibroid was soft, round and hernia-like with rich blood supply. The myoma was completely resected by hysteroscopy, and all symptoms disappeared.The ultrasound examination found no abnormality 1-month post-surgery. During follow-up, ultrasound examination revealed a low echo mass with rich blood supply located in left intramural myometrium walls where the previous operation site was, and gradually increased form approximately 2.5 to 4 cm in diameter at 5 and 8 months.Until this admission, we gave her GNRH-a injection 3 times(3.75 mg/28days), but the mass grew out of the uterus,growing along the left ovarian vein reaching the level of the lumbar 5-sacral 1 disc, and ultrasound venography indicated it’s intravenous leiomyomatosis(IVL). Echocardiography and computed tomography venography (CTV) of inferior vena cava were performed and showed heart and other blood vessels with no abnormality. Laparoscopic bilateral salpingo-oophorectomy and hysterectomyis were scheduled.

**Fig. 1:**
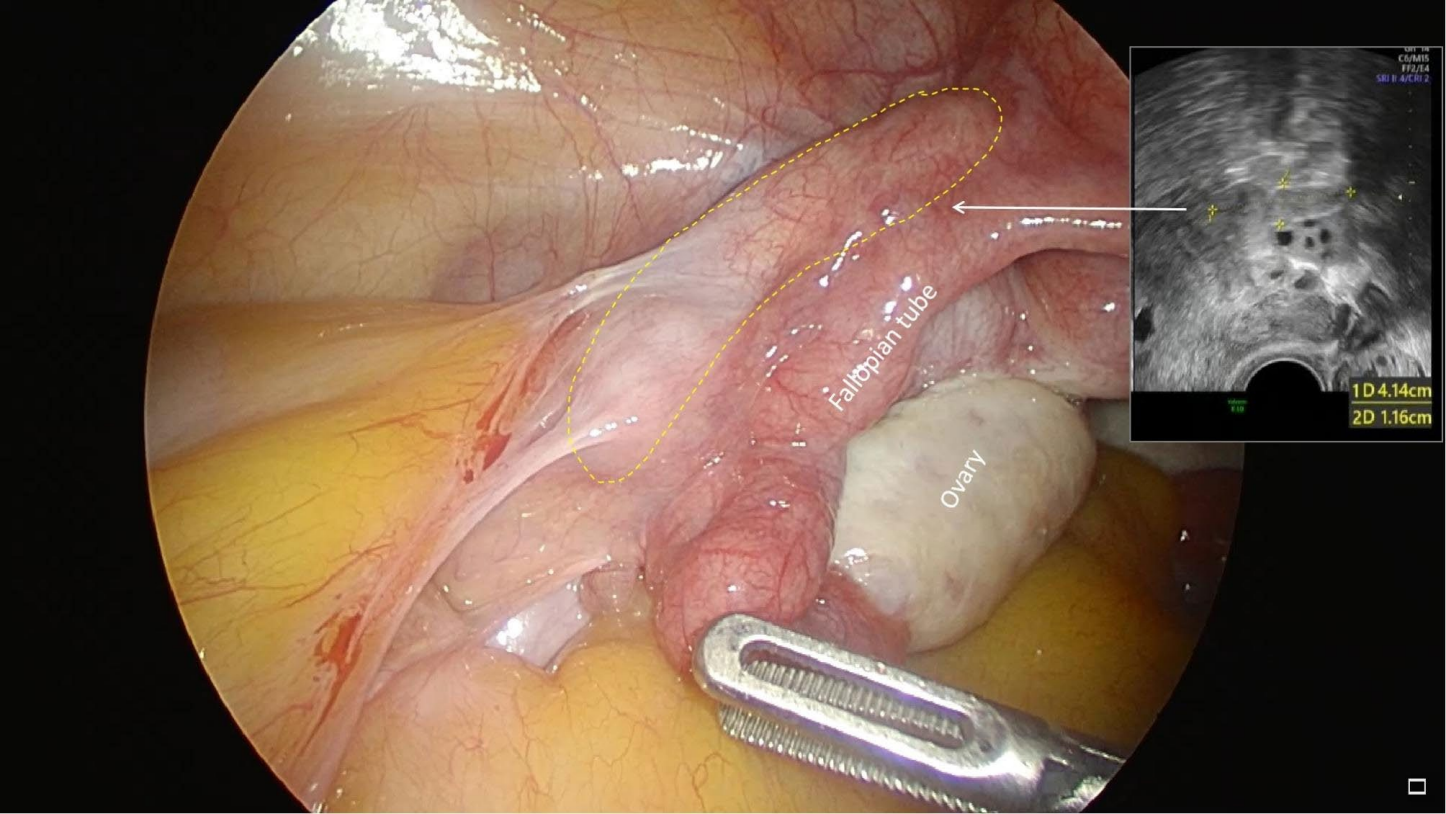
The IVL in the left ovarian vein

**Fig. 2:**
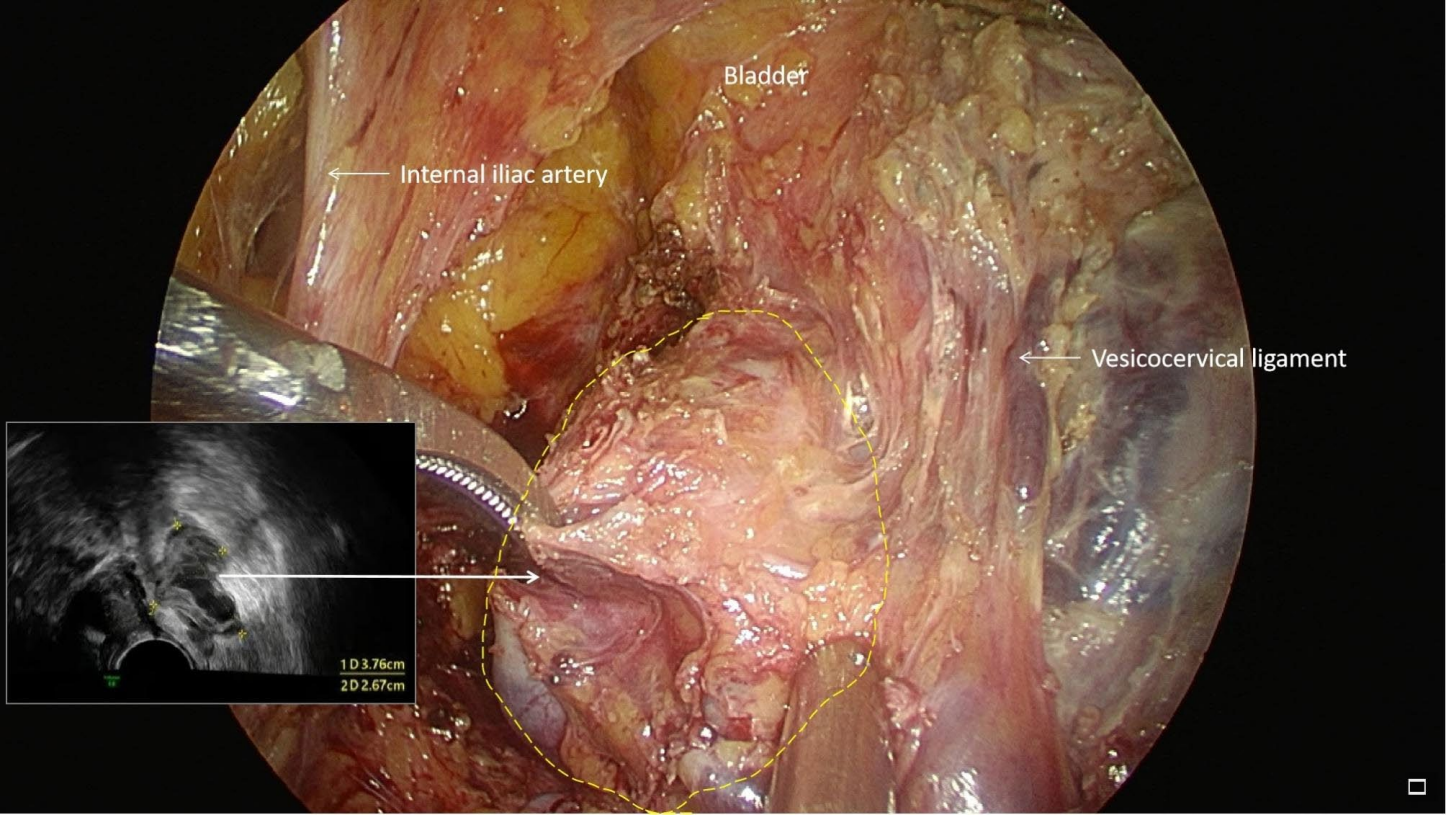
The IVL in the left parauterine venous plexus

**Fig. 3:**
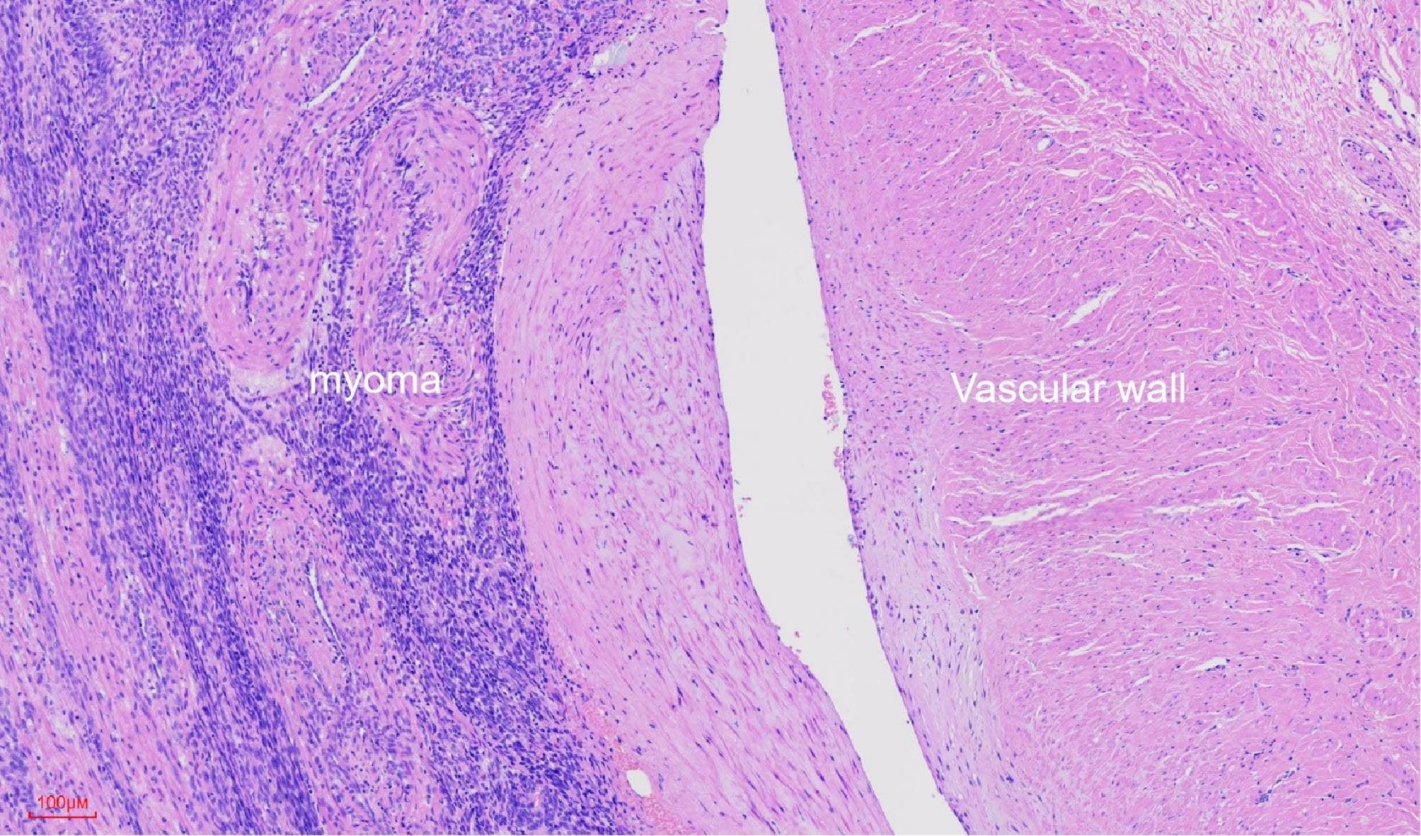
Histopathological results confirmed intravenous leiomyomatosis

During this surgical operation, the uterus looked normal size, with a uneven surface, and the cord-like tissues in the left ovarian vessel was detected. During the hysterectomy, many rope and mass-like muscle tissues surrounding with abnormal proliferation blood vessels in the left inferior uterine body to the cervix were observed, and was closely related to the left ureter and the left lateral wall of the bladder.The IVL in the left ovarian vein and parauterine venous plexus were excised completely during surgery(Fig. 1 Fig. 2 ). The post-operative histopathological results confirmed uterine leiomyoma and IVL (Fig. 3). The lesions of IVL was completely resected by laparoscopy, thereby successfully blocking the progression of the lesions to cardio-pulmonary vascular which may endanger the patient’s life.The patient recovered well after surgery.No surgical-related or anesthesia-related complications occurred.The 3-month follow-up CTV of inferior vena cava and echocardiography examination revealed normal.

## Discussion

Intravascular leiomyomatosis (IVL) is a histologically-benign, rare mesenchymal tumor which can develop from the veins of the uterus, extend into the inferior vena cava and ultimately into the right side of the heart, resulting in death.The aetiology of IVL is unclear and discussion centres around two hypothesis, namely,the proliferation of vascular smooth muscle cells and the direct invasion of uterine leiomyoma into the muscular veins and diffusion along the vascular lumen [6, 7].According to the reported cases, about 83. 5-94.3% of IVL patients had uterine fibroids or previous history of uterine fibroid surgery as reported [4, 5], so most scholars believe that IVL is caused by uterine leiomyoma directly invading the veins of myometrium. Chen et al. analyzed the clinical data of 361 IVL patients(38 patients from Qilu Hospital of Shandong University and 323 patients from the published literature) and confirmed the hypothesis that IVL originates from the uterus to a certain extent [8]. Similarly, Van et al. found that the estrogen and progesterone receptor in IVL cells were weakly positive to strongly positive, while normal vascular smooth muscle cells were often negative or weakly positive, also confirming that IVL originated in the uterus [9]. The patient we report here obtained IVL after hysteroscopic myomatectomy. In addition, imaging examination, intraoperative exploration and postoperative pathology all indicated that IVL was connected to the uterus, which also supported this theory. Therefore, we have reason to believe that our patient’s IVL was derived from a previous submucosal myoma.

At present, the management guidelines for IVL is unavailable, and the current treatment is mainly based on limited case reports. According to previous reports, the postoperative recurrence rate is high after incomplete tumor resection, approximately 16.6–30.0% [10]. The preferred treatment for IVL is surgery with radical tumour resection to reduce the risk of disease progression and future recurrence [11]. The specific surgical strategies and approaches should be combined with the staging and invasion range of the tumor. In our patient, the tumor was found to grow upward along the left ovarian vein during follow-up, and the patient received laparoscopic surgery timely. The IVL in the left ovarian vein and parauterine venous plexus were detected and excised completely during surgery. The lesions of IVL was completely resected by laparoscopy, thereby successfully blocking the progression of the lesions to cardio-pulmonary vascular which may endanger the patient’s life.The patient recovered well after surgery.No new lesions were found during the 3-month follow-up.

## Conclusions

We report a case of IVL that progressed after submucosal myomectomy. The cause of IVL is unknown, this observation demonstrates that hysteroscopic myomectomy might lead to the occurrence of IVL. Due to the rarity of IVL, most current research is limited to case reports. Nevertheless, case report is limited by the quality of information available. Systematic literature review or multi-center studies about IVL is beneficial to increased awareness on the etiology and treatment of IVL. In addition, up to now, the present case has been followed up for only several months and reported no recurrence. It is possibly because the follow-up time was short, which needs to continue for life. Early detection and accurate diagnosis are imperative for appropriate treatment.

## Data Availability

All data generated or analyzed during this study are included in this published article.

## Abbreviations

IVL: intravenous leiomyomatosis
CTV: Computed tomography venography
